# Adjuvanted Modified Bacterial Antigens for Single-Dose Vaccines

**DOI:** 10.3390/ijms252111461

**Published:** 2024-10-25

**Authors:** Roberta Di Benedetto, Luisa Massai, Mark Wright, Francesca Mancini, Matthew Cleveland, Omar Rossi, Carlo Giannelli, Francesco Berlanda Scorza, Francesca Micoli

**Affiliations:** 1 GSK Vaccines Institute for Global Health (GVGH), 53100 Siena, Italy; roberta.x.di-benedetto@gsk.com (R.D.B.); luisa.x.massai@gsk.com (L.M.); francesca.x.mancini@gsk.com (F.M.); omar.x.rossi@gsk.com (O.R.); carlo.x.giannelli@gsk.com (C.G.); francesco.x.berlandascorza@gsk.com (F.B.S.); 2 GSK, Stevenage SG1 2NFX, Hertfordshire, UK; c-mark-wright@outlook.com (M.W.); mattcle@googlemail.com (M.C.)

**Keywords:** aluminium hydroxide, alum, vaccines, recombinant proteins, glycoconjugates, single dose

## Abstract

Alum is the most used vaccine adjuvant, due to its safety, low cost and adjuvanticity to various antigens. However, the mechanism of action of alum is complex and not yet fully understood, and the immune responses elicited can be weak and antigen-dependent. While several antigens rapidly desorb from alum upon exposure to serum, phosphorylated proteins remain tightly bound through a ligand-exchange reaction with surface hydroxyls on alum. Here, bacterial proteins and glycoconjugates have been modified with phosphoserines, aiming at enhancing the binding to alum and prolonging their bioavailability. Tetanus toxoid protein and *Salmonella* Typhi fragmented Vi-CRM conjugate were used. Both antigens rapidly and completely desorbed from alum after incubation with serum, verified via a competitive ELISA assay, and set up to rapidly evaluate in vitro antigen desorption from alum. After antigen modification with phosphoserines, desorption from alum was slowed down, and modified antigens demonstrated more prolonged retention at the injection sites through in vivo optical imaging in mice. Both modified antigens elicited stronger immune responses in mice, after a single injection only, compared to unmodified antigens. A stronger binding to alum could result in potent single-dose vaccine candidates and opens the possibility to design novel carrier proteins for glycoconjugates and improved versions of bacterial recombinant proteins.

## 1. Introduction

Vaccination is one of the most efficient strategies for controlling human infectious diseases globally. Vaccine efficacy depends not only on the antigen components, but also on the adjuvant used. Indeed, adjuvants can be defined as components that enhance the immunogenicity of vaccines when administered in conjunction with the antigens [1]. Moreover, independently of their mechanism of action, the use of adjuvants can lead to (I) the reduction in antigen amount per vaccine dose and the number of vaccinations required to induce protective immunity, (II) a more rapid induction of protective response, and (III) the increase in seroconversion rates in populations with reduced responsiveness, e.g., because of age, disease, or therapeutic interventions [2,3,4].

Aluminium hydroxide (alum) is the most commonly used vaccine adjuvant due to the good safety profile, low cost, and adjuvanticity provided to a variety of antigens. However, the mechanism of action of aluminium adjuvants is complex and not yet fully understood. It likely involves various mechanisms acting simultaneously, including (I) the formation of a depot, (II) the efficient uptake of aluminium-adsorbed antigens by antigen-presenting cells due the particulate nature of alum formulation, and (III) its optimal size for stimulation of immune competent cells of the body [5,6,7]. Although effective for many vaccines, immune responses elicited by alum formulations are, in other cases, weak and antigen-dependent. Nevertheless, given the long-term success of aluminium adjuvants, they continue to represent an important gold-standard benchmark for the development of new adjuvants [6]. Preparation of alum-formulated vaccines involves the adsorption of antigens to aluminium gels. However, many antigens are rapidly desorbed from alum upon exposure to serum or interstitial fluid [8,9,10].

Recently, Moyer et al. proposed site-specific modification of viral antigens with short peptides composed of repeating phosphoserines (pSer) as a way to enhance binding to alum and prolong immunogen bioavailability [11]. Indeed, phosphorylated proteins are known to be much more tightly bound to alum than antigens relying solely on electrostatic or hydrogen-binding interactions, through a ligand exchange reaction with surface hydroxyls on alum [12,13]. The pSer-modified antigens formulated in alum promoted quantitative and qualitative improvements in the humoral and cellular immune responses elicited [11].

In this study, a similar approach was tested for the first time with bacterial antigens, with the aim to investigate possible methods to enable the development of potent and long-lasting single-dose vaccines, which can be crucial in instances of a pandemic, for maternal immunisation and for Low- and Middle-income Countries (LMICs). We started with tetanus toxoid (TT), a vaccine that is clinically effective against tetanus firstly produced in 1924 [14] and also extensively used as carrier protein for glycoconjugate vaccines [15]. Moreover, TT is already known to rapidly and completely desorb from alum at the injection site [16]. Then, we extended the approach to glycoconjugates, selecting *Salmonella* Typhi fragmented Vi-CRM (fVi-CRM) as the model [17,18].

Here, the TT and fVi-CRM antigens were modified with a pSer linker and compared to corresponding unmodified antigens via a competitive enzyme-linked immunosorbent assay (competitive ELISA, cELISA), set up to rapidly evaluate in vitro the degree of antigen adsorption to alum after incubation with a certain percentage of serum. Moreover, all the formulations were tested in mice, and the induced humoral immune response was investigated together with antigens clearance at the injection site using in vivo optical imaging.

Results from this work further confirm that a stronger binding to alum could result in more potent vaccine candidates and open the possibility of designing novel carrier proteins for glycoconjugates and improved versions of bacterial recombinant proteins.

## 2. Results

### 2.1. TT Modified with Phosphoserine Linker Exhibits More Stable Binding to Alum and Prolonged Retention at the Injection Site

A phosphoserine linker constituting of four consecutive pSer, a short poly(ethylene glycol) spacer and a *N*-terminal maleimide functional group (pSer4) [11] was used to introduce phosphate groups onto the TT protein, in order to investigate if and how this modification could impact the binding of this antigen to alum and, consequently, its immune response.

**Figure 1:**
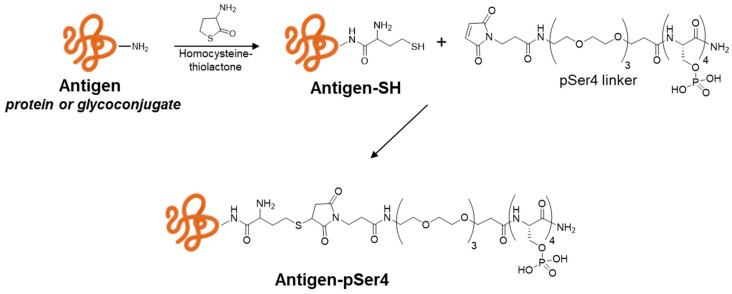
Bacterial protein and glycoconjugate antigen derivatisation with the pSer4 linker using thiol-maleimido chemistry. Activation of the aminogroups on the protein or glycoconjugate with *N*-acetyl-DL-homocysteine thiolactone, introducing thiol groups which then react with the maleimide group of the pSer4 linker.

The TT derivatisation with pSer4 was performed via thiol-maleimido chemistry. TT was first activated with *N*-acetyl-DL-homocysteine thiolactone, targeting aminogroups on the protein, in order to introduce the thiol groups, which then could react with the pSer4 linker, terminating in a maleimide group (Figure 1). On average, five linkers were introduced as verified via matrix-assisted laser desorption/ionisation (MALDI) mass analysis. The absence of free linkers after purification and protein integrity was verified via size-exclusion high-performance liquid chromatography (HPLC-SEC).

TT and pSer4-modified TT (TT-pSer4) were investigated for their desorption from alum after exposure to serum. A cELISA-based assay was set up with this purpose (Figure S1). Both antigens were adsorbed on alum and incubated with 10% mouse serum, and the concentration of not-adsorbed antigen, before and after incubation with the serum, was measured by cELISA. TT antigens formulated without alum, at the same protein concentration with respect to the alum-adsorbed ones, were added as positive controls.

**Figure 2:**
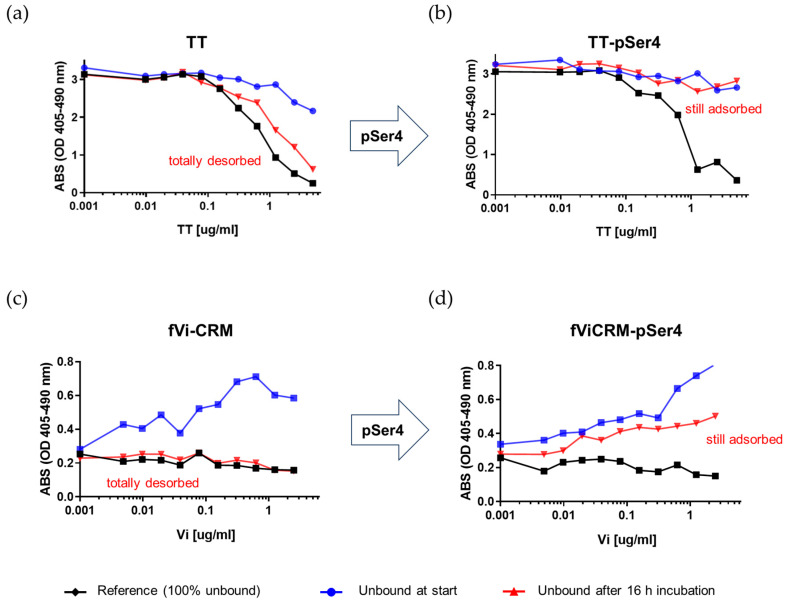
cELISA was used to evaluate, in vitro, the binding stability of TT (**a**), TT-pSer4 (**b**), fVi-CRM (**c**) and fVi-CRM-pSer4 (**d**) to alum. Antigens were formulated without alum (Reference) at a concentration of 10 µg/mL (TT and TT-pSer4) or 5 µg/mL (fVi-CRM and fVi-CRM-pSer4, Vi-based). The same antigens were formulated with alum (0.7 mg/mL Al^3+^) at the same concentrations and corresponding supernatants after centrifugation were collected at time 0 and after 16 h incubation with 10% mouse serum. All samples were compared in their ability to compete for anti-TT (**a**,**b**) or anti Vi (**c**,**d**) antibody binding, measuring the amount of unbound antigen. In each graph, curves of the antigen without alum (reference corresponding to 100% unbound antigen with alum, black line), the supernatant at time 0 (blue line) and the supernatant after incubation with serum (red line) are reported. After incubation with serum, the antigen was totally desorbed for TT and fVi-CRM (red line overlapping black line), and still adsorbed for TT-pSer4 and fVi-CRM-pSer4 (red line overlapping blue line).

When the alum-formulated TT was incubated with 10% mouse serum, the toxoid completely desorbed after 16 h of incubation (Figure 2a), confirming what has already been published for this antigen [16]. Similar results were obtained after 10 min of incubation with the serum. The introduction of pSer4 linkers altered the alum-adsorption profile of TT, achieving stable binding after 16 h of incubation with 10% mouse serum (Figure 2b). Similar results were also obtained using 20% mouse serum.

**Figure 3:**
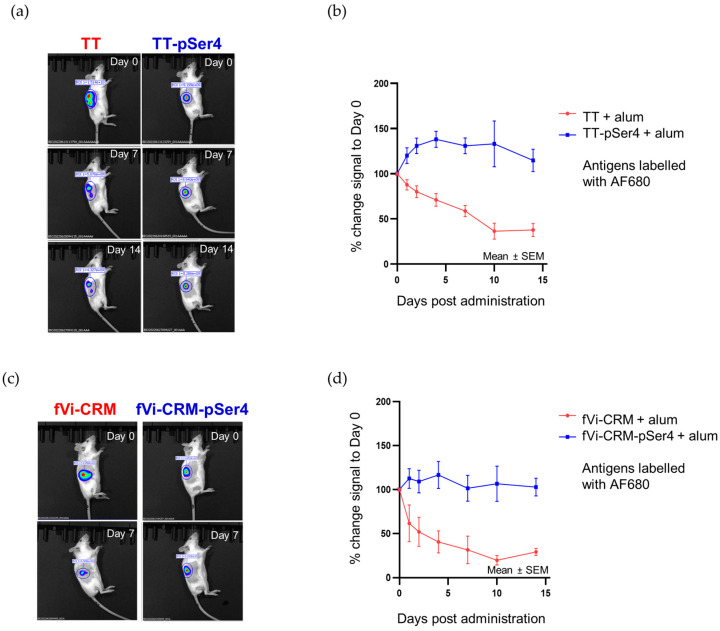
CD1 mice were immunised subcutaneously (s.c.) with a single injection of TT, TT-pSer4 labelled with AF680 (5 µg TT/dose) (**a**,**b**) or fVi-CRM and fVi-CRM-pSer4 labelled with AF680 (5 µg Vi/dose) (**c**,**d**), all formulated with alum (0.7 mg/mL Al^3+^). Biodistribution in mice was tracked by in vivo optical imaging. Fluorescence images at the injection sites were acquired at approximately 30 min post immunisation and at days 1, 2, 4, 7, 10, and 14 post immunisation. Example images (**a**,**c**) and % change of signal detected from day 0 from groups of animals over time (**b**,**d**) are reported. The centre lines represent the mean, while the error bars represent the standard error of the mean (SEM).

To evaluate the in vivo biodistribution characteristics of the pSer4-modified TT, mice were injected with a single dose of TT or TT-pSer4 labelled with Alexa Fluor 680 (AF680). No impact of the presence of AF680 on the antigens’ alum-adsorption profiles was verified via cELISA, for both TT (Figure S2a,b) and TT-pSer4 (Figure S2c,d), prior to immunisation. Fluorescence at the injection site was tracked using in vivo optical imaging. Unmodified TT demonstrated signal decay from the injection site over the time course (within 14 days after immunisation), whereas TT modified with pSer4 demonstrated prolonged retention at the injection site (Figure 3a,b).

### 2.2. TT-pSer4 Elicits Higher Immune Response in Mice After One Injection Compared to Unmodified TT

**Figure 4:**
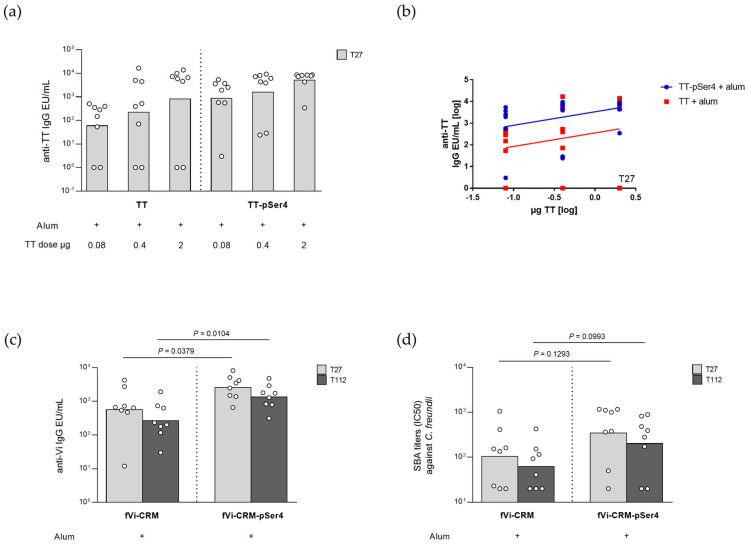
Immunogenicity in mice immunised with (**a**,**b**) TT and TT-pSer4 or (**c**,**d**) fVi-CRM and fVi-CRM-pSer4. CD1 mice were immunised (**a**,**b**) intraperitoneally (i.p.) on day 0 with 0.08, 0.4 and 2 µg TT/dose or (**c**,**d**) s.c. at day 0 with 0.2 µg Vi/dose. All constructs were formulated with alum at 0.7 mg/mL Al^3+^. Sera were analysed for (**a**) anti-TT- or (**c**) anti-Vi-specific IgG EU/mL by ELISA, and for (**d**) bactericidal titres expressed as IC50 by serum bactericidal activity (SBA) assay. Summary graphs of geometric mean units (bars) and individual levels (dots) are reported. (**b**) Parallel line analysis shows dose–response curves at day 27 of the two TT formulations (log-transformed IgG EU/mL titres in the Y axis and log-transformed TT doses in the X axis).

The immunogenicity of TT and corresponding pSer4-modified construct were compared in mice. In the absence of alum, the proteins were tested at a dose of 2 µg TT. No impact of the derivatisation chemistry used to insert the pSer4 linker onto the toxoid was verified on the induced anti-TT total IgG response, both 27 days after the first immunisation and 14 days post second dose (Figure S3a). In the presence of alum, both antigens were instead tested at three different doses, ranging from 0.08 to 2 µg of TT. Mice were immunised once, and the response was evaluated 27 days after the injection (Figure 4a). Modified TT elicited a stronger immune response after only one injection than the corresponding unmodified protein, in the dose range tested. Indeed, comparing the dose–response curves at day 27, unmodified TT induced a significantly lower IgG response than -pSer4, with a potency of 4.1% (95% confidence interval 0–54.6%) (Figure 4b).

### 2.3. Extending the Method to Glycoconjugate Vaccines: Linkage of pSer4 to fVi-CRM Increases Binding Stability to Alum and Retention at the Injection Site

We decided to extend the method to glycoconjugate vaccines and verify whether the pSer modification could be applied to a more complex bacterial vaccine. Using *Salmonella* Typhi fVi-CRM conjugate, a rapid and total desorption of the antigen from alum was also observed in this case after incubation with mouse serum (Figure 2c).

The pSer4 linker was introduced to fVi-CRM (fVi-CRM-pSer4) to alter the stability of antigen binding to alum and verify if this could be translated in improvements in the immune response induced. The same chemistry used for TT was applied directly to fVi-CRM, targeting the aminogroups on the carrier protein. Overall, 37% of the -NH_2_ groups were activated, as verified by 2,4,6-trinitrobenzene 1-sulfonic acid assay (TNBS) [19] (Figure 1). The absence of free linker after purification was verified via HPLC-SEC. Differently from TT, the average number of pSer4 linkers on the conjugate was not determined, due to the low ionisation efficiency of MALDI with glycans. However, the presence of pSer4 linkers on fVi-CRM was confirmed by cELISA, as the alum-adsorption profile of fVi-CRM was altered, with binding to alum retained after 16 h of incubation with 10% (Figure 2d) or 20% mouse serum.

Both fVi-CRM and fVi-CRM-pSer4 were labelled with AF680, verifying that there was no impact on the antigens’ alum-adsorption profiles through cELISA (Figure S4). Again, mice were injected with a single dose of labelled fVi-CRM or fVi-CRM-pSer4, to evaluate the clearance from the injection site over time through in vivo optical imaging. Similarly to what was already seen with TT, for fVi-CRM, signal decay from the injection site was detected, while for fVi-CRM derivatised with pSer4, the signal remained high over the time course investigated (Figure 3c,d).

### 2.4. fVi-CRM-pSer4 Induces a Stronger Immune Response in Mice After One Injection Also at Later Timepoints

The immunogenicity of fVi-CRM, with and without phosphoserine groups, was compared in mice. Without alum, no impact of the derivatisation chemistry used to modify the conjugate was verified, obtaining similar anti-Vi total IgG responses 27 days after one dose and 2 weeks after a second injection (Figure S3b). With alum, the two antigens were tested with a dose of 0.2 µg Vi, selected based on previous dose ranging studies performed [18]. Mice were injected only once, and the response was not only evaluated 27 days after the immunisation, but also after 4 months to look at the antibodies’ persistency. Similarly to that observed with TT, the pSer-modified construct induced a higher Vi-specific IgG response with respect to the unmodified fVi-CRM, 27 days after the injection (Figure 4c). Moreover, the response elicited by fVi-CRM-pSer4 remained significantly higher compared to fVi-CRM also at the latest timepoint evaluated (112 days after immunisation) (Figure 4c). Sera functionality was tested by serum bactericidal activity (SBA) against a *Citrobacter freundii* strain which expresses the Vi antigen [20,21]. The sera induced by both constructs were functional. The trend was the same as that observed for ELISA, but no statistically significant differences were highlighted (Figure 4d).

## 3. Discussion

In this work, we have tested the modification of bacterial antigens with phosphoserine linkers as a method for the development of potent and long-lasting single-dose alum-adjuvanted vaccines. Typically, multiple doses of the same vaccine are required to reach an adequate level of protection, but the development of vaccines inducing protective immunity with fewer doses, or, ideally, just one, would be advantageous. Single-dose vaccines would be optimal to maximise vaccination coverage, decrease the costs associated with multi-dose regimens, and improve patient convenience. In particular, this strategy would be attractive for LMICs and mass vaccination programs to combat future pandemics [22,23].

It is, nowadays, widely accepted that prolonged antigen exposure could lead to strong immune responses, with qualitative and quantitative improvements [24,25,26,27], which might be necessary for the design of potent single-dose vaccines.

The use of adjuvants is one of the approaches available to modulate and promote antigen availability after vaccination [22]. Certain adjuvants may form an antigen depot at the site of injection, facilitating the slow release of antigens after vaccination. This was one the mechanisms hypothesised for alum [28,29], even if this adjuvant actually exerts several other effects on the immune system [7,30,31,32,33]. A depot mechanism of action has been also hypothesised for other newer adjuvants, such as the oil-in-water emulsion MF59 [28,34]; the cationic adjuvant formulations (CAF platform), containing the surfactant dioctadecylammonium (DDA), formulated into liposomes or emulsions [35,36,37]; and the IC31 adjuvant, which is an agonist of the Toll-like receptor (TLR)-9 [38,39].

Despite the availability of several new adjuvants, alum remains the most used for licensed vaccines. It is safe, tolerable, and its production is cost-effective. Moreover, its use, being an established adjuvant, would facilitate both clinical development and regulatory pathways [6].

A recent paper from Moyer et al. has reported that if the absorption of the antigen on alum is increased—through chemical modification of viral antigens with short peptides composed of repeating pSer residues—this may result in the enhancement of germinal centres and antibody responses, shaping both the quantity and the quality of the induced immune response [11].

For the first time, this approach has been extended to bacterial antigens, namely TT protein and fVi-CRM glycoconjugate, modified with pSer residues, with the aim to evaluate the impact of this modification on the humoral immune responses induced, possibly resulting in single-dose vaccines. A novel competitive ELISA-based in vitro assay has been developed, allowing us to rapidly evaluate the strength of the linkage between antigens and alum after incubation with serum. Indeed, it is known that following exposure to interstitial fluid, most antigens are rapidly desorbed from aluminium adjuvants [40]. Using this assay, it was confirmed that, after the introduction of the pSer linker, both the TT and fVi-CRM degrees of desorption from alum were slowed down with respect to the unmodified antigens. Importantly, this was correlated in vivo with augmented retention of the pSer-antigens at the injection sites, as verified up to 14 days post injection via optical imaging. Moving forward, for each specific antigen, it will be important to check whether depot formation at the injection site can impact local inflammation response and innate immune system activation.

In terms of the humoral immune response induced, after a single injection only, both phosphorylated antigens elicited a stronger total IgG response than that induced by the corresponding unmodified ones. This was particularly evident for TT-pSer4. For fVi-CRM-pSer4, the IgG response was higher and persisted at higher levels for 112 days after the immunisation compared to fVi-CRM, while sera functionality remained similar.

For both antigens we verified, in the absence of alum, no impact of the chemistry used to introduce the pSer linker to the specific immune response was induced. This is a key point, as in other studies with phosphorylated antigens, it was shown that increasing the strength of the antigen binding to alum had no or even a negative impact on the magnitude of the IgG response induced [8,12,41,42]. This may be due to the random modification of protein antigens over the entire protein surface with alum-binding sites, which could allow the immunogen to crosslink alum particles together or become denatured upon binding, either of which could have unpredictable effects on the immune response [11]. In our case, particularly for the fVi-CRM glycoconjugate, targeting the carrier protein for derivatisation allowed us to entirely overcome this issue, leaving the saccharide component untouched. Also, for TT, which was directly targeted with the derivatisation, the level of modification was controlled as not to alter the antigen-specific immune response induced. In the future, recombinant techniques may be investigated to directly obtain phosphorylated counterparts of the desired antigens, with a specific number of phosphate groups avoiding the use of linkers and derivatisation chemistries, trying to modulate adsorption on alum, and further verifying its impact on the immune response.

## 4. Materials and Methods

### 4.1. Antigens

TT protein was obtained from GSK R&D (Siena, Italy).

The fVi-CRM conjugate was produced and purified as previously described [18].

### 4.2. Synthesis of pSer4-Antigens via Thiol-Maleimido Chemistry

TT and fVi-CRM were first activated with -SH linker (*N*-acetyl-DL-homocysteine thiolactone); both antigens were diluted to have a final antigen concentration of 5 mg/mL in PBS. The activation buffer containing 2.6 mg/mL Dithiothreitol (DTT), 13.16 mg/mL Ethylenediaminetetracetic acid (EDTA), and 7.04 mg/mL *N*-acetyl-DL-homocysteine thiolactone in 100 mM borate buffer at pH 11 was added, in order to reach a 1:1 molar ratio of thiolactone to -NH_2_ groups. The reaction was mixed at room temperature (RT) for 4 h, at the end of which the purification was carried out using prepacked single-use PD10 columns (Cytiva Life Sciences, Marlborough, MA, USA) against 10 mM 2-(*N*-morpholino)ethanesulfonic acid (MES) buffer at pH 6 and 1 mM EDTA.

The TT and fVi-CRM antigens activated with -SH were then added to the pSer4 linker having a terminal maleimide functional group, in a 4:1 molar ratio of pSer4 to -SH groups. The pSer4 linker (constituted by 4 consecutive phosphoserines, a short poly(ethylene glycol) spacer and the *N*-terminal maleimide functional group) was purchased as a custom peptide from Merck, Darmstadt, Germany. The reaction was mixed at RT overnight, and the purification was carried out using PD10 columns against PBS.

### 4.3. Antigens Labeling with Alexa Fluor 680 (AF680) Succinimidyl Ester (NHS Ester)

TT and fVi-CRM, with and without pSer4, were labelled with AF680 dye as follows: The antigens were buffer-exchanged against 100 mM sodium bicarbonate at pH 8.3 through Amicon Ultra (Merck, Darmstadt, Germany) 30 kDa cut-off (3500 rpm; 20 °C; 2 washes of 15 min). Immediately before use, the succinimidyl ester was dissolved in dimethylsulfoxide (DMSO) at 10 mg/mL and added to the antigen in a 10:1 (for TT) or 5:1 (for fVi-CRM) molar ratio of dye to antigen (protein-based). The reaction was mixed at RT for 2 h, and the purification was carried out through Amicon Ultra 30 kDa cut-off against PBS (3500 rpm; 20 °C; 6 washes of 15 min).

### 4.4. Analytical Characterisation

The total protein concentration in TT and fVi-CRM antigens was measured by micro Bicinchoninic acid (BCA) (Thermo Scientific, Waltham, MA, USA); the total Vi content was measured by high-performance anion-exchange chromatography with pulsed amperometric detection (HPAEC-PAD) [43].

The introduction of average numbers of thiolactone and pSer4 linkers in TT was measured via MALDI [44]. The activation of aminogroups on fVi-CRM with thiolactone was quantified via TNBS assay [19]. The absence of free pSer4 linker in derivatised TT and fVi-CRM after purification was verified through HPLC-SEC analysis [45].

Antigen labelling with AF680 was confirmed by HPLC-SEC [45] comparing the fluorescence of the labelled and native antigens, in fluorescence emission detection, using ex684-em707.

### 4.5. Preparation of Formulations

TT proteins (0.4–25 µg/mL) and fVi-CRM glycoconjugates (0.5–25 µg/mL Vi) were adsorbed on alum at the final concentration of 0.7 mg/mL Al^3+^ or diluted in saline buffer without any adjuvant.

### 4.6. cElisa-Based Assay to Evaluate Antigens Adsorption to Alum After Incubation with Serum

Each antigen was formulated with and without alum at the same antigen concentration, ranging from 5 to 10 µg/mL. The antigen without alum was the reference, corresponding to 100% not-adsorbed. Half of the antigen formulated with alum was immediately centrifuged for 5 min at 14,000 rpm, and then incubated with 10% *v*/*v* preimmune mouse serum (unbound at time 0); the other half was first incubated with 10% *v*/*v* preimmune mouse serum and, after 16 h, centrifuged for 5 min at 14,000 rpm (unbound after incubation with 10% serum).

The cELISA was performed using round-bottom Nunc Maxisorp ELISA plates (Thermo Scientific, Waltham, MA, USA) coated overnight at 4 °C with 100 μL/well of TT or fVi at 1 µg/mL in PBS, and blocked the following day with 200 µL/well of 5% fat-free milk dissolved in PBS buffer at 25 °C for 1 h. Then, plates were washed three times with 250 µL of washing buffer (PBS with 0.05% Tween-20 (PBS-T). Competitive ELISA was set up using anti-Tetanus Toxoid antibody [3F6] (Abcam, Cambridge, UK) and anti-Vi (Denka, Tokio, Japan) as primary antibodies. The antibody solutions were spiked (ratio 1:1) with serial dilutions of the samples. The mixtures prepared in PBS-Tween 0.05% and BSA 1% were incubated for 2 h at 25 °C on the coated ELISA plates, with samples and coating competing for the binding to the primary antibody. The stronger the binding between the primary antibody and the antigens present in the sample solution, the weaker the binding to the coated antigen; hence, the lowest signal will be obtained, and vice versa. Plates were washed three times with 250 µL of washing buffer (PBS with 0.05% Tween-20 (PBS-T), and then 100 µL of secondary anti-mouse or anti-rabbit IgG conjugated to alkaline phosphatase (Merck, Darmstadt, Germany) were added to the plates and incubated for 1 h at 25 °C. After three additional washes in PBS-T, 100 µL of p-nitrophenyl phosphate substrate solution (Merck, Darmstadt, Germany) was added, and plates were incubated for 1 h at 25 °C. Absorbances were read with an automatic plate reader (Biotek, Winooski, VT, USA) at 405 nm and subtracted of the background at 490 nm.

### 4.7. Animal Studies and Assessment of Antibody Responses

GSK is committed to the Replacement, Reduction, and Refinement of animal studies (3Rs). Non-animal models and alternative technologies are part of our strategy and employed where possible. When animals are required, the application of robust study design principles and peer review minimises animal use, reduces harm, and improves benefit in studies.

TT and fVi-CRM immunogenicity studies in mice were performed at the Charles River Laboratories facility (Chatillon, France), in accordance with the European Directive 63/2010. The animal protocols were approved as part of the GSK internal ethical review process by the internal Animal Welfare Body (Project No. R-0245, approval date 1 January 2021).

CD1 female mice, 5 weeks old (8 per group), were vaccinated intraperitoneally (i.p.) or subcutaneously (s.c.) with 200 µL of antigens at study days 0 and 28, or at day 0 only. Approximately 100 µL bleeds (50 µL serum) were collected at day -1 (pooled sera) and at day 27 (individual sera), with the final bleed at either day 42 or at day 112.

The imaging study was ethically approved as part of the GSK internal ethical review process (Home Office project licence P174E12D2, review date 1 March 2022), performed at facilities at GSK Stevenage (UK), were carried out in accordance with the Animals (Scientific Procedures) Act 1986 and the GSK Policy on the Care, Welfare and Treatment of Laboratory Animals.

CD1 female mice, 5–6 weeks old (8 per group), were immunised s.c. with 200 µL of antigens at study day 0. Two days before the vaccination, mice were close-shaved in the right flank to aid image acquisition and to prepare the injection site. Images were acquired at approximately 30 min post immunisation and at days 1, 2, 4, 7, 10, and 14.

Mouse sera were analysed for anti-TT and anti-Vi total IgG by enzyme-linked immunosorbent assay (ELISA), as previously described, using TT and Vi (in phosphate buffer, at the concentration of 1 µg /mL) as coating antigens [45,46].

### 4.8. High-Throughput Serum Bactericidal Activity Assay

Individual serum samples collected at day 27 and 112 from mice immunised with fVi-CRM constructs were also tested against the *Citrobacter freundii* 3056 strain in SBA based on luminescent readout as previously described [20,47]. Briefly, the *Citrobacter* strain from frozen glycerol stock was grown in Luria-Bertani medium to log phase (0.18–0.25 OD600) and then diluted to approximately 1 x 106 CFU/mL in PBS. Diluted bacteria, heat-inactivated sera, and exogenous BRC were mixed and incubated for 180 min at 37 °C. At the end of the incubation, the plate containing the assay reaction was centrifuged at 25 °C, and RT for 10 min at 4000× *g*. The supernatant was discarded to remove all bacterial debris, and the bacterial pellet was resuspended in PBS, transferred to white round-bottom 96-well plates (Greiner Bio-One, Stonehouse, UK), and mixed 1:1 (*v*:*v*) with BacTiter-Glo Reagent (Promega, Madison, WI, USA). After 5 min of incubation at RT on an orbital shaker, the luminescence signal was measured by a luminometer (Synergy Biotek, Winooski, VT, USA). Bactericidal serum titres were calculated as the reciprocal serum dilution necessary to obtain 50% bacterial growth inhibition.

### 4.9. In Vivo Optical Imaging

For the first image acquisition, the mice were anaesthetised by approved protocols (induction at 5% isoflurane in oxygen/air) prior to immunisation with fluorescently labelled antigen. The mice were then monitored for any adverse events (none observed), before being transferred to the IVIS manifold (IVIS Spectrum, Revvity Inc., Waltham, MA, USA), where they were kept under isoflurane anaesthesia (2–3% isoflurane in oxygen/air) and kept warm on a heated stage. For subsequent image acquisition, the mice were anaesthetised and imaged without being monitored. Photographic and fluorescent images were captured using the acquisition parameters set out for Alexa Fluor 680 fluorophore within the Living Image software (version 4.5, Revvity Inc., Waltham, MA, USA).

All quantifications of regions of interest (ROI) were generated using the Living Image software according to the manufacturer’s instructions (Revvity Inc., Waltham, MA, USA). Briefly, fluorescence signal intensity was manually identified in each mouse by an operator. A single ROI larger than the visual boundaries of the fluorescence signal was defined to ensure that the entire area of signal diffusion was included in each measurement. The same ROI was used across all images acquired for that corresponding mouse.

### 4.10. Statistics

Statistical analysis was performed using GraphPad Prism 7. The Mann–Whitney two-tailed test was used to compare the immune response elicited by two different formulations. The Wilcoxon matched-pairs signed-rank two-tailed test was used to compare the response induced by the same formulation at different timepoints.

Dose–response curve (ln dose vs. ln EU) comparisons were performed using CombiStats Software version 7.0 (EDQM, Strasbourg, France). The parallelism of the lines was tested first by comparison of the slopes. If this resulted in not-significant difference, subsequently, the Y-intercepts of the curves obtained were compared. The results of the analysis are reported as potency values with their confidence interval.

## 5. Conclusions

In conclusion, our work confirms, along with bacterial antigens, that a stronger binding to alum could result in more potent single-dose vaccine candidates, and opens the possibility for designing novel carrier proteins for glycoconjugates and improved versions of bacterial recombinant proteins.

## Data Availability

The authors declare that the data are contained within the article and in the Supplementary Materials.

## Supplementary Materials

The following supporting information can be downloaded at: https://www.mdpi.com/article/10.3390/ijms252111461/s1.
