# Supplementary material for: Adjuvanted Modified Bacterial Antigens for Single-Dose Vaccines
Source: Int J Mol Sci. 2024 Oct 25;25(21):11461. doi: 10.3390/ijms252111461 (PMC11546299; doi:10.3390/ijms252111461)
Supplement: Supplementary file 1 [file ijms-25-11461-s001.zip › ijms-3267024-supplementary.pdf]

Supplementary Figures

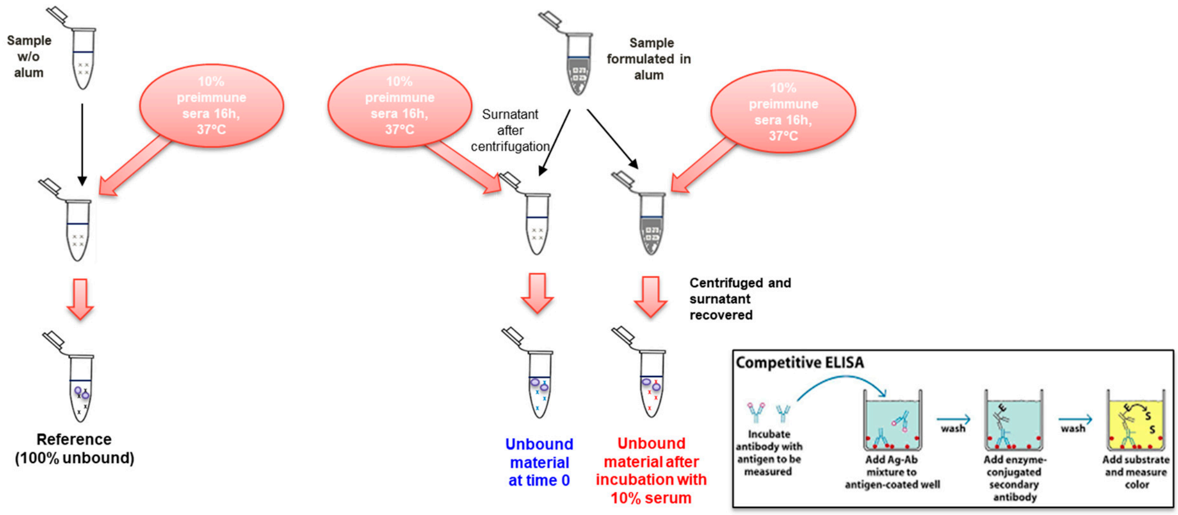

Figure S1. cELISA-based assay used to evaluate antigens binding stability to alum after incubation with 10% mouse serum. Each antigen was formulated with and without alum at the same concentration. The antigen without alum (reference at 100% unbound), the antigen with alum centrifuged before (unbound at time 0) or after incubation with mouse serum (unbound after incubation with 10% serum) were then compared in their ability to compete for anti-specific antigen antibodies binding.

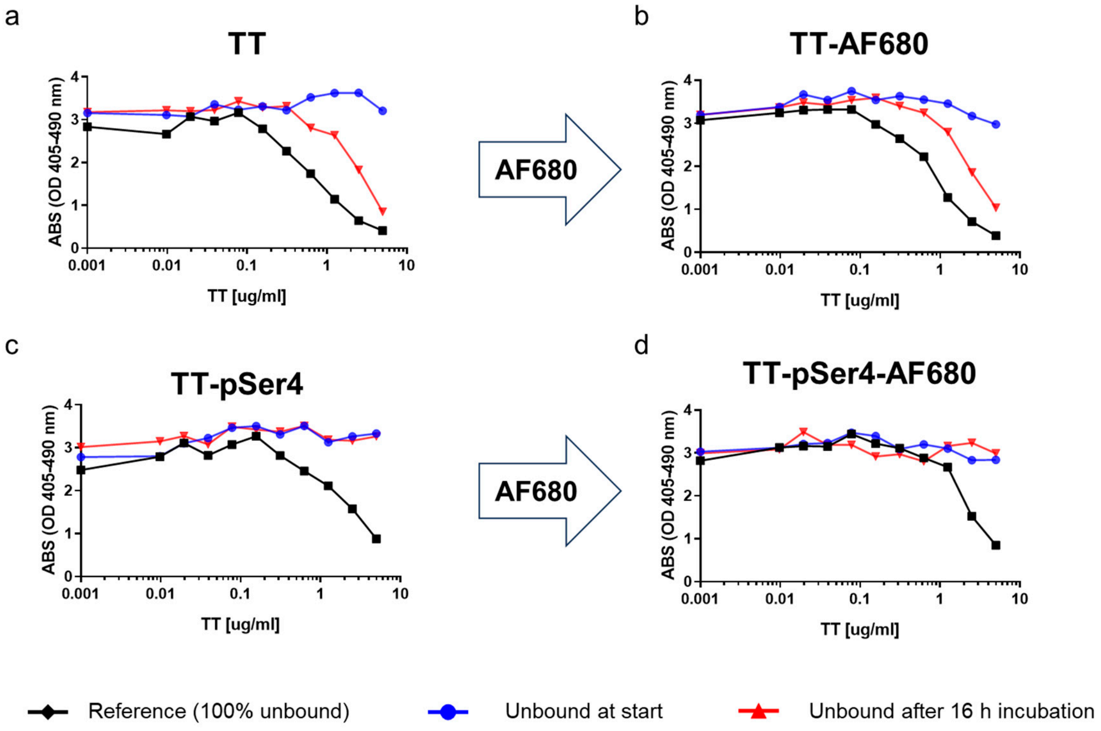

Figure S2. cELISA used to evaluate *in vitro* TT (a), TT-AF680 (b), TT-pSer4 (c) and TT-pSer4-AF680 (d) binding stability to alum after 16 h of incubation with 10% mouse serum. All proteins were formulated with alum (0.7 mg/mL Al^{3+}) and without alum at the same concentration of 10 μg/mL. In each graph: the protein without alum (reference at 100% unbound, black line), the supernatant after centrifugation of the protein with alum at time 0 (unbound at start, blue line) and the supernatant after centrifugation of the protein with alum incubated with 10% serum for 16 h (unbound after incubation with serum, red line) were compared in their ability to compete for anti-TT antibodies binding.

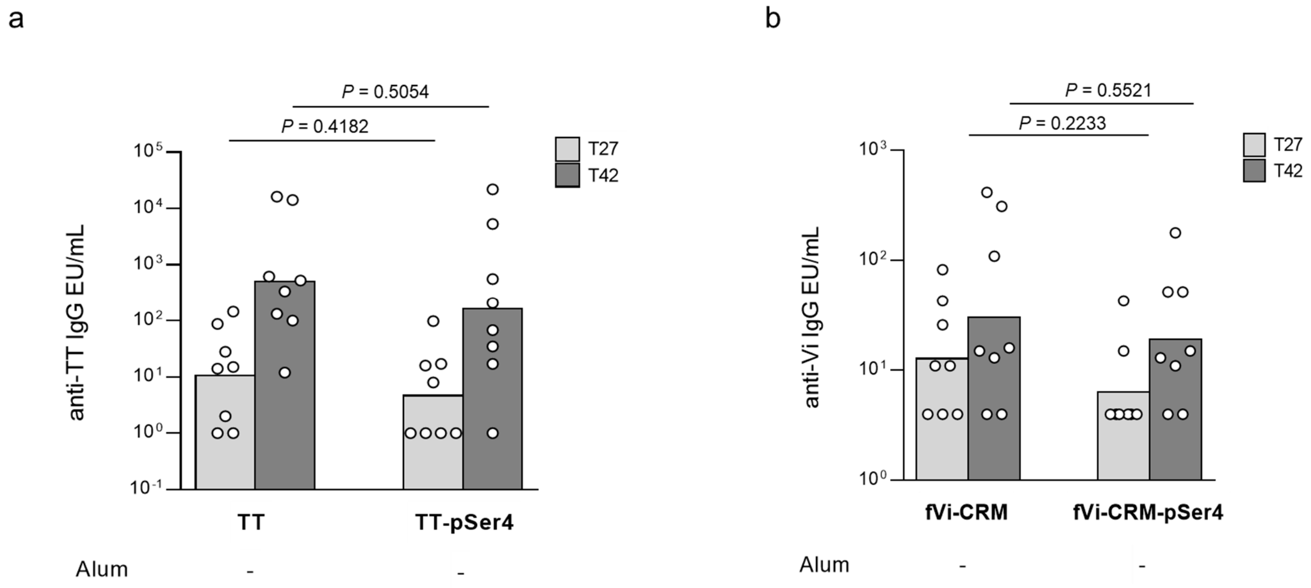

Figure S3. Immunogenicity in mice of (a) TT and TT-pSer4 or (b) fVi-CRM and fVi-CRM-pSer4 without alum. CD1 mice were immunized intraperitoneally (i.p.) at day 0 and 28 with (a) 2 µg TT/dose or (b) 0.1 µg Vi/dose. Summary graphs of (a) anti-TT and (b) anti-Vi specific IgG EU/mL geometric means (bars) and individual antibody levels (dots) are reported.

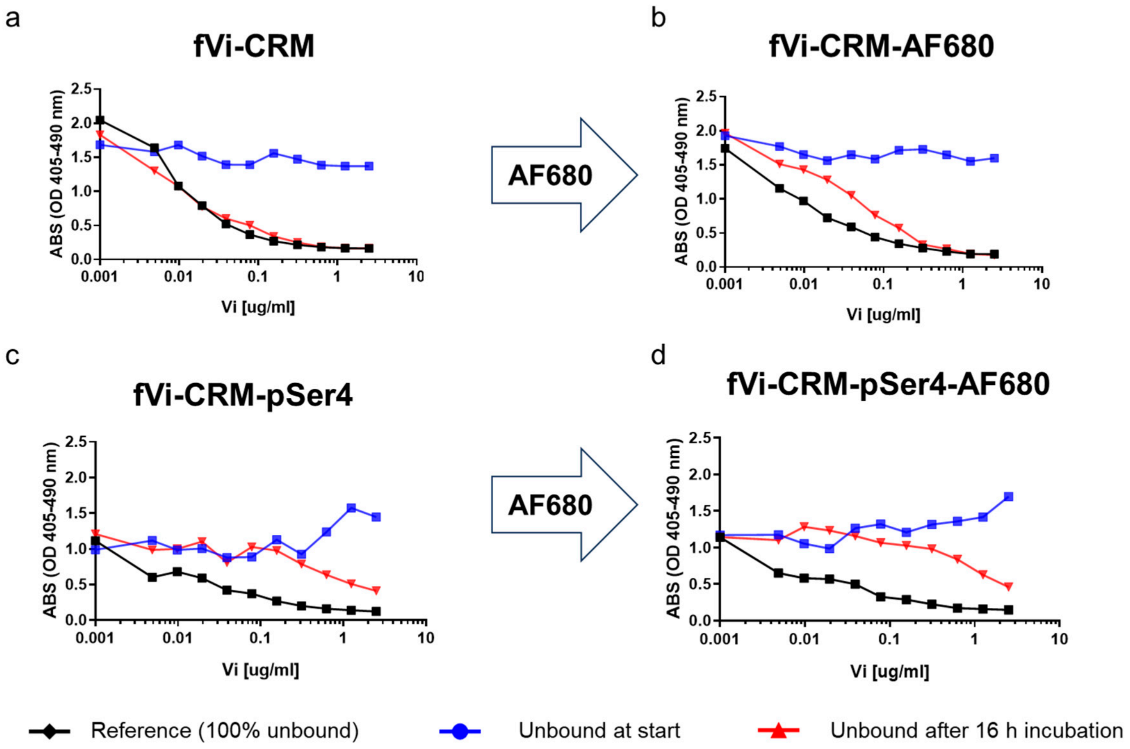

Figure S4. cELISA used to evaluate *in vitro* fVi-CRM (a), fVi-CRM-AF680 (b), fVi-CRM-pSer4 (c) and fVi-CRM-pSer4-AF680 (d) binding stability to alum after 16 h of incubation with 10% mouse serum. All conjugates were formulated with alum (0.7 mg/mL Al^{3+}) and without alum at the same concentration of 5 µg/mL Vi-based. In each graph: the conjugate without alum (reference at 100% unbound, black line), the supernatant after centrifugation of the conjugate with alum at time 0 (unbound at start, blue line) and the supernatant after centrifugation of the conjugate with alum incubated with 10% serum for 16 h (unbound after incubation with serum, red line) were compared in their ability to compete for anti-Vi antibodies binding.
